# Single-Cell Probe Force Studies to Identify Sox2 Overexpression-Promoted Cell Adhesion in MCF7 Breast Cancer Cells

**DOI:** 10.3390/cells9040935

**Published:** 2020-04-10

**Authors:** Jagoba Iturri, Andreas Weber, María d.M. Vivanco, José L. Toca-Herrera

**Affiliations:** 1 Department of Nanobiotechnology (DNBT), Institute for Biophysics, BOKU University for Natural Resources and Life Sciences, Muthgasse 11 (Simon Zeisel Haus), A-1190 Vienna, Austria; andreas.weber@boku.ac.at (A.W.); jose.toca-herrera@boku.ac.at (J.L.T.-H.); 2 Cancer Heterogeneity Lab, Center for Cooperative Research in Biosciences (CIC bioGUNE), Basque Research and Technology Alliance (BRTA), Bizkaia Technology Park, 48160 Derio, Spain

**Keywords:** single-cell probe, AFM, epifluorescence, TIRF, cell adhesion, MCF7 cells, Sox2 overexpression

## Abstract

The replacement of the cantilever tip by a living cell in Atomic Force Microscopy (AFM) experiments permits the direct quantification of cell–substrate and cell–cell adhesion forces. This single-cell probe force measurement technique, when complemented by microscopy, allows controlled manipulation of the cell with defined location at the area of interest. In this work, a setup based on two glass half-slides, a non-fouling one with bacterial S-layer protein SbpA from *L. sphaericus* CMM 2177 and the second with a fibronectin layer, has been employed to measure the adhesion of MCF7 breast cancer cells to fibronectin films (using SbpA as control) and to other cells (symmetric vs. asymmetric systems). The measurements aimed to characterize and compare the adhesion capacities of parental cells and cells overexpressing the embryonic transcription factor Sox2, which have a higher capacity for invasion and are more resistant to endocrine therapy in vivo. Together with the use of fluorescence techniques (epifluorescence, Total Internal Fluorescence Microscopy (TIRF)), the visualization of vinculin and actin distribution in cells in contact with fibronectin surfaces is enabled, facilitating the monitoring and quantification of the formation of adhesion complexes. These findings demonstrate the strength of this combined approach to assess and compare the adhesion properties of cell lines and to illustrate the heterogeneity of adhesive strength found in breast cancer cells.

## 1. Introduction

Cancer-induced alterations at the molecular level result in changes at the cellular and tissue level, leading to defects in cell proliferation, migration, and adhesion. Cell adhesion is mediated in cell–cell and cell–extracellular matrix (ECM) interactions by adhesion receptors, such as cadherins, integrins, and proteoglycans. The tight control of cell biochemical and mechanical microenvironment through ECM components like fibronectin, collagen, proteoglycans or laminin is disturbed during the various phases of cancer disease [1]. This leads to ECM remodeling accompanied by increased fiber alignment and matrix stiffening [2]. Adhesion dysregulation is crucial for cancer dissemination and the establishment of metastasis in other tissues with vastly different microenvironments. The process starts with cancer cell detachment, the passing of cellular barriers, transport through a fluid system, and finally adhesion and proliferation at a distant site [3,4]. Therefore, an improved understanding of the interactions of cancer cells with ECM components is warranted.

Studying the interplay between cells and ECM components requires elucidation of surface properties. Engineering matrix protein scaffolds can significantly alter the attachment of cells on surfaces, presenting an environment to the cells that is closer to the situation in vivo [5,6]. Two-dimensional surface modifications are sensed by cells and transduced via multiple signaling pathways, which influence cell motility, proliferation, and mechanical properties [7,8,9]. The application of thin coatings of fibronectin (FN) to substrates is widely used as cell culture technique to improve cell adhesion. Fibronectin is a large ECM glycoprotein that carries binding sites for both integrins as well as syndecans [10]. A complex interplay of groups of receptor molecules is needed to promote the formation of focal adhesions, strengthen the actin cytoskeleton, and induce cell spreading [11,12].

The characterization of cell adhesion to substrates in vitro facilitates the elucidation of the underlying molecular mechanisms. Cellular attachment and spreading on surfaces are governed by reactions of different force and time scales that need to be considered. To date, a large variety of techniques has been developed to study adhesion. Still widely in use are cell attachment and cell spreading assays, centrifugal assays, and optical microscopy techniques, such as bright field, fluorescence microscopy, or interferometry. In recent years, surface sensitive techniques such as quartz crystal microbalance (QCM) or surface plasmon resonance (SPR) have been developed, as well as various passive (including particle displacement, particle deformations due to strain, stress-based FRET sensors, etc.) and active (including atomic force microscopy (AFM) and optical and magnetic tweezers) measurement techniques [13,14]. Of those techniques, AFM offers a unique combination of scale (nm to µm) and force (pN to µN), while enabling measurements in ambient conditions, (bio)chemical modifications of the probe, and combination with optical microscopy [15,16,17,18]. AFM cantilevers can be chemically modified to pick up cells that are loosely attached to the underlying substrate. Thus, cells are used to probe the interaction with either surfaces or other cells, permitting force-, position-, and time-resolved adhesion measurements [19,20]. Recently, we have successfully demonstrated an experimental approach to prepare cell samples for such measurements utilizing bacterial surface layer protein coatings, known by their high efficiency as anti-fouling coatings [21,22,23], in a feasible, easy-to-handle, one-step manner [24].

The altered expression profiles of ECM components, such as integrins, cadherins, and syndecans, have been found to correlate with tumorigenesis, tissue invasiveness, metastatic behavior, and survival in many types of cancer, including breast cancer [25,26]. Breast cancer presents the highest incidence of all tumors and is still the first cause of death from cancer in women. Approximately 70% of breast tumors express the estrogen receptor (referred to as ER-positive) and, therefore, they are treated with endocrine therapy. Tamoxifen, an ER antagonist, has successfully been used in the clinic for many years; however, very often resistance to therapy develops [27]. Various mechanisms have been revealed to underlie development of resistance to therapy, including the presence of cells with stem-like properties. Cancer stem-like cells (CSCs) have been shown to be implicated in tumor initiation and resistance to different forms of therapy [28]. We have found that tamoxifen-resistant cells are enriched for CSCs and express high levels of the stem cell marker Sox2 [29], which is also expressed in normal breast stem cells, although at lower levels than in breast tumor cells [30,31]. The ectopic expression of Sox2 reduces tamoxifen sensitivity in vitro and in vivo and tamoxifen-resistant cells display increased invasion ability [29]. Furthermore, Sox2 increases the expression of another transcription factor, Sox9, which is also implicated in maintenance of luminal progenitor and breast CSCs [29,32]. Other reports have correlated high levels of Sox2 expression with increased survival, self-renewal capacity, and metastatic outgrowth in other types of tumors as well [33,34,35,36]. We hypothesized that the overexpression of Sox2 leads to changes in the expression of proteins related to breast cancer cell adhesion.

To analyze the alterations on cell adhesion, single-cell probe AFM force–distance measurements were performed to determine the adhesion strength of parental and Sox2 overexpressing MCF7 cells, quantifying cell-fibronectin, as well as cell–cell interactions. Through variation in cell–surface and cell–cell contact time, we were able to study the attractive interaction in a time-dependent manner. For fibronectin surfaces, the maximum strength of adhesion was significantly highest for Sox2 cells. In turn, the adhesion work was highest for MCF7 cells. This reveals the difference in the adhesion pattern of both cells. Cell–cell adhesion was similar for homotypic interactions and decreased two- to three-fold for asymmetric measurements, highlighting the influence of adhesion heterogeneity within breast tumors.

## 2. Materials and Methods

### 2.1. S-Layer Protein Preparation

The bacterial cell–surface layer protein SbpA (Mw = 120 kDa) was isolated from *L. sphaericus* CMM 2177 following the standard protocol. Briefly, the extraction process was achieved by guanidine hydrochloride (5 mM) followed by dialysis for 2 h against deionized water, which reduces the chaotropic reagent (guanidine hydrochloride) to a concentration of 0.2 to 0.5 mM. After isolation, the protein solution was centrifuged for 5 min in order to separate the S-protein monomers from self-assembly products and was stored at 4 °C. Protein recrystallization buffer was prepared with 0.5 mM Trizma base (Merck KGaA, Darmstadt, Germany) and 10 mM CaCl_2_ (Merck KGaA, Darmstadt, Germany) and was adjusted to pH 9. Before each experiment, the supernatant solution was diluted using the appropriate amount of recrystallization buffer to a final concentration of 0.1 mg/mL (ca. 85 mM).

### 2.2. Sample Functionalization

Borosilicate circular cover glasses (diameter: 24 mm, thickness: 0.08–0.12 mm, Menzel Gläser, VWR, Bruchsal, Germany) were cut into two pieces, rinsed with EtOH, N_2_ dried, and cleaned with oxygen plasma (GaLa Instrumente GmbH, Bad Schwalbach, Germany) prior to functionalization. Each glass piece was immersed into the desired coating solution: 20 µg/mL of bovine fibronectin (FN, Merck KGaA, Darmstadt, Germany) in phosphate buffered saline (PBS) buffer or 0.1 mg/mL of SbpA protein in recrystallization buffer. The coating time of fibronectin samples was 1 h, whereas SbpA samples required overnight incubation. Incubations took place at room temperature (RT). Substrates were then carefully cleaned with MilliQ water before each experiment in order to remove remaining materials.

### 2.3. Cantilever Functionalization

Tipless silicon nitride cantilevers with a nominal stiffness of 0.12 N/m (NP-0, Bruker) were cleaned under oxygen plasma. Freshly cleaned cantilevers were immersed into a drop of 1 mg/mL poly-L-lysine (PLL) solution for 1 h at room temperature and were subsequently cleaned with ultrapure water. Further functionalization was carried out by immersing PLL-cantilevers into a drop of 20 µg/mL fibronectin solution for 1 h at room temperature. Fibronectin-coated cantilevers were stored into Milli-Q water until use.

### 2.4. Cell Culture and Sample Preparation 

MCF7 cells were obtained from the American Type Culture Collection (ATCC). Sox2 over-expressing cells were obtained using lentiviral transduction, as previously reported [30]. Cells were seeded in 75 cm^2^ flasks using Dulbecco’s Modified Eagle Medium (Gibco, Thermo Fisher Scientific, Waltham, MA USA) supplemented with 10% Fetal Bovine Serum (FBS) (Gibco) and 1% penicillin/streptomycin. Sox2 overexpressing cells were treated with puromycin (5 µL from 1 mg/mL solution in water per 10 mL) in order to keep the selection pressure. The cells were cultured at 37 °C, with 5% CO_2_. Immediately before experiments, the cell layer was dispersed using 2 mL of TrypLE^TM^ Express (Gibco) and was then centrifuged, counted, and redispersed in Leibovitz’s L-15 medium (Gibco). Cells (1 × 10^5^) in suspension were used for AFM measurements. They were injected into the measuring setup and left for sedimentation for 30 min.

### 2.5. Atomic Force Microscopy (AFM)

**Figure 1:**
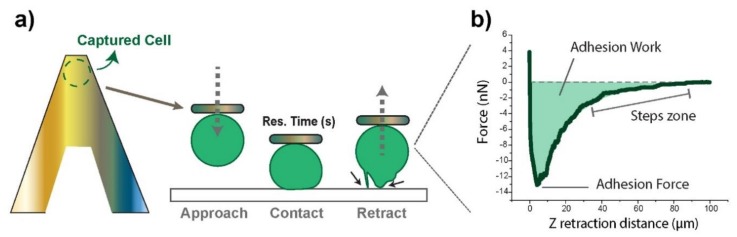
(**a**) Schematic drawing of a cell positioned onto a poly-L-lysine (PLL)-fibronectin functionalized cantilever, and a description of a usual Force experiment. The black arrows point at different adhesion events as the cell is detached from the surface. The inset on the right-hand side (**b**) shows a real retraction force–distance curve derived from such an experiment, following the conditions described at the materials and methods section after 90 s of contact. The analyzed parameters are highlighted graphically: the maximum adhesive force represents the minimum in the force curve, the work of adhesion the area under the curve, and the steps the gradual zero-force recovery pattern.

Measurements were performed in cell medium environment at 37 °C by using the JPK custom thermo-regulated flow-cell. Functionalized cantilevers were calibrated before each experiment by means of the thermal tune method. The spring constant of the tipless cantilever was around 0.12 N/m. AFM instrument JPK Nanowizard III (Bruker, Berlin, Germany) with CellHesion mounted on an inverted optical microscope (Axio Observer Z1, Zeiss, Jena, Germany) was operated, after the attachment of the cell, in force-spectroscopy mode. Experimental settings involved constant force (3 nN), constant approach (5 μm/s) and retract (10 μm/s) rates, and varying residence times (0–120 s) or the amount of time the cell stayed in contact. A schematic description of the experiments performed is shown in Figure 1a. Measurements were performed either on different substrates (SbpA or fibronectin) or on other cells (symmetric or asymmetric) since the Z height of the piezo has a range of 100 μm. Individual cells were adhered onto the PLL-fibronectin cantilever by employment of the Cell Capture mode by JPK software, over cells standing on the S-layer side (see Supplementary, Figure S1).

Experiments exclusively focused on the retraction segment, which allowed for obtaining adhesion-related factors (Figure 1b) as the probing cell was moved away (Z retraction distance). These included the maximum adhesion force required to break the contact (minimum in the curve) and the adhesion work required until complete detachment (F = 0), given by the area under the curve. The stepwise recovery trend indicated gradual contact loss. Adhesion Forces appear as negative by definition, since starting positive values correspond to the setpoint force. The axes’ origin (0,0) refers to null cantilever deflection in the retracting motion.

### 2.6. Cell Fixation, Permeabilization, and Immunostaining

The cells were grown for 24 h at a concentration of 5 × 10^4^ cells/mL on fibronectin-coated (20 µg/mL) Ibidi µ-slides using the above specified media. The cells were fixed with 4% paraformaldehyde for 10 min at RT. Then, cells were permeabilized using 0.1% Triton X-100 (diluted in PBS) at RT for 15 min and blocked using 2% BSA (in PBS) at RT for 1 h. For immunostaining, primary rabbit anti-Vinculin antibody diluted in 1% BSA in PBS (dilution 1:125) was incubated at 4 °C overnight. The secondary antibody (goat anti rabbit IgG, diluted in 1% BSA in PBS, 1:500) conjugated with Alexa Fluor 633 was added and incubated for 45 min at RT together with fluorescently labeled phalloidin (Alexa Fluor 555), to stain the actin filaments (diluted in 1% BSA in PBS, 1:40). Finally, the nuclei were stained using Hoechst (diluted in 1% BSA in PBS, 1:1000 of stock). For TIRF measurements, anti-rabbit Abberior STAR 512 was used as a secondary antibody for vinculin staining (1:50). The samples were kept at 4 °C protected from light and measured as soon as possible. At least two independent samples (with the respective controls) were produced. All materials were purchased from Thermo Fisher Scientific (Waltham, MA, USA).

### 2.7. Epifluorescence Microscopy and Total Internal Fluorescence Microscopy (TIRF)

For epifluorescence studies, a Leica DMI6000B wide-field fluorescence microscope with a white light lamp (EL6000) was used. A 40× oil immersion objective was employed. Images were taken at different optical planes with optimized illumination settings. For TIRF, a similar microscope stand was used, equipped with lasers at 405, 488, 563, and 635 nm as well as the appropriate filters. A 100× oil immersion objective was employed. After the calibration and determination of the surface region, a TIRF field of either 90 or 220 nm (near to epifluorescence) was employed to image different cellular regions. The images were processed using the Fiji distribution of ImageJ.

## 3. Results

### 3.1. Cell–Substrate Interactions

#### 3.1.1. Strength and Work of Adhesion Differ for MCF7 and Sox2 Overexpressing Cells

**Figure 2:**
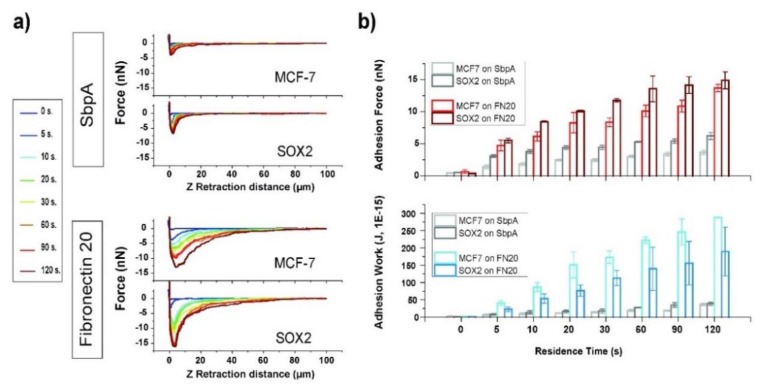
Cell–substrate interactions. (**a**) The resulting force–distance plots for the two cell types when placed in contact with the corresponding substrate (SbpA vs. fibronectin). (**b**) The column plots obtained for adhesion force (in nN) and the work of adhesion (in J, 1E-15) for the respective cases under study.

In Figure 2a, representative force–distance curves for control (MCF7) and Sox2-overexpressing (Sox2, see Supporting Information, Figure S2a) MCF7 cells in contact with fibronectin and SbpA substrates are shown upon variation of the contact time from 0 to 120 s. The attractive interaction between the cells and the S-layer surface appears to be quite low, as expected considering the previously proven anti-fouling capability of such films. Sox2 overexpressing cells had higher affinity for the SbpA coating at longer residence times than MCF7 cells. The adhesion between cells and fibronectin-coated substrate was significantly stronger than on the S-layer. Qualitatively, the force–distance curves show differing adhesion behavior: for Sox2 overexpressing cells, a defined adhesion peak with fast recovery of zero-force can be seen, while the peak appears to be broadened and smaller for MCF7 cells (Figure 2a, lower panel), suggesting that the maximum adhesive force was highest in cells with elevated levels of Sox2. 

The interaction was enhanced with an increasing time of contact (Figure 2b and Supplementary Table S1). The maximum adhesion force increased significantly for all studied systems when extending the contact time up to 120 s. For the fibronectin system, a contact time of 5 s led to a ten-fold increase in the resulting adhesion force (from 0.5 to 5 nN). The adhesion force increased following a linear trend for MCF7 cells from 20 to 120 s of contact time. Sox2 cells showed a bimodal rise with two linear regimes, one up to 60 s and the second one with a decreased slope up to 120 s (Supplementary Information, Figure S2b). For intermediate contact times, the adhesion force appears to be highest for Sox2 cells, suggesting differences in the kinetics for the formation of adhesion complexes. After 120 s of contact with the substrate, no significant differences were detectable between the two cell lines.

Similarly, the adhesive work was increased in a time dependent manner (Figure 2b, lower panel). MCF7 cells showed higher adhesion work for all studied cell–substrate contact times. The difference between both cell lines was most significant at a contact time of 20 s, with the work for Sox2 cells being almost two-fold (150 vs. 76 fJ). These values are in good agreement with previous findings in literature [37,38]. Further investigation of the curve shape was performed by fitting exponential decay functions to the recovery to zero-fore. While a single-exponential decay was suited to describe the behavior of MCF7 cells, for Sox2 overexpressing ones, a second order decay function fitted better (Supplementary Figure S3, Table S2), thus underlining the differing behavior of Sox2 overexpressing cells versus parental cells.

#### 3.1.2. Sox2 Overexpression Leads to Increased Membrane–Cytoskeleton Connection

**Figure 3:**
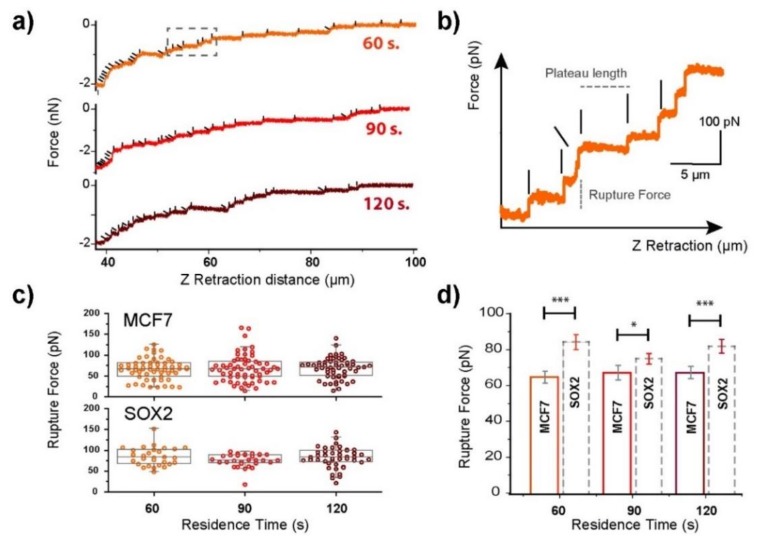
Contact time dependence on the tether formation for cells in contact with fibronectin (20 µg/mL) surfaces. (**a**) Representative tether recording at three different contact times for MCF7 cells, and (**b**) the corresponding magnified view from the area highlighted by a dashed rectangle. The plateau length and rupture force factors are indicated in the plot. The vertical and tilted black lines highlight the position of single-step and intermediate rupture events, respectively. (**c**) Rupture force distributions. The horizontal line represents the median and the values range from the 5th to the 95th quantile. (**d**) Mean rupture force calculation for both control (MCF7), and Sox2 overexpressing cells. The significance of the variations in the *p* < 0.05 and *p* < 0.005 level is indicated by * and *** accordingly.

Step-like ruptures were present in the retraction curves, corresponding to the rupture of membrane tethers between cell and substrate (Figure 1). The retract plots of cells adhering to fibronectin films after residence times of 60, 90, and 120 s were obtained (Figure 3). The analysis was set to above 40 µm since at this point the events could be easily discriminated from each other. Below 40 µm, the presence of a large number of small ruptures was identified, but it was extremely difficult to resolve them. Individual force jumps for MCF7 cell ruptures were recorded (Figure 3a, highlighted by black lines, where the tilted ones refer to short intermediate steps). A magnified view on the kind of recorded rupture events is shown in Figure 3b, where the two factors to consider are indicated: the plateau length (distance between jumps) and the rupture force, which represents the force needed to break one bond or rupture the contact with a membrane tether. The rupture force is a parameter employed to investigate membrane fluidity and the connection of the membrane to the cytoskeleton [39,40]. A looser connection between both leads to a reduction in the force needed to rupture such a tether. A lower number of rupture events in the range >40 µm from the cell was recorded for Sox2 overexpressing cells compared to MCF7 (Figure 3c). The distribution of plateau lengths (data not shown) was similar for both cell lines. The rupture force distribution for MCF7 cells (Figure 3c) appeared more spread than for Sox2 overexpressing cells. For the studied contact times, the average rupture force per tether was significantly higher for Sox2 overexpressing cells with an approximate ratio of 1:1.2. These observations suggest that the membrane–cytoskeleton connection is tighter in Sox2 than in parental MCF-7 cells. 

#### 3.1.3. Soluble Fibronectin Decreases Adhesion in Both Systems

**Figure 4:**
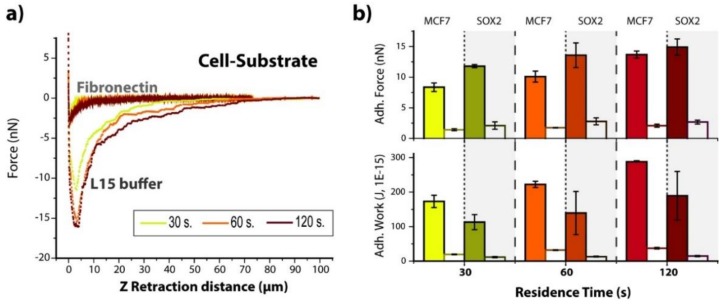
Influence of soluble fibronectin. (**a**) Representative force–distance plots for Sox2 cells interacting with Fibronectin substrates in L15 medium either with or without fibronectin addition, and increasing contact times (30, 60 and 120 s). (**b**) Calculated values of maximum adhesion force and adhesion work for both MCF7 and Sox2 cells before (filled columns, values from Figure 2) and after (empty columns) the injection of soluble fibronectin.

The role of adhesion complex–fibronectin interactions on the adhesion of MCF7 and Sox2 overexpressing cells to the substrate was studied by measurements in the presence of soluble fibronectin (2 µg/mL) at three contact times (30, 60 and 120 s) (Figure 4 and Supplementary Table S3). The adhesion peak decreased drastically in the presence of soluble fibronectin (Figure 4a,b). In general, adhesion forces suffered a 5- to 6-fold decrease, while final adhesion work values were 10% of the starting ones (in L15 medium). Interestingly, comparison of Sox2 and MCF7 cells still showed a similar behavior to measurements in L15 medium with a higher adhesion force for Sox2 but a larger adhesion work for MCF7 cells. These findings suggest that the cell–surface interactions might be driven by other mechanisms. This would require the performance of a deeper study in the future.

### 3.2. Asymmetric Cell–Cell Adhesion is Reduced

Single-cell probe force measurements enable the study of interactions between different cells. Thus, both symmetric (MCF7–MCF7 and Sox2–Sox2), as well as asymmetric (MCF7–Sox2) systems, were investigated. Nucleus staining of Sox2 overexpressing cells was performed for identification between cells under asymmetric systems (Supplementary Figure S4). 

**Figure 5:**
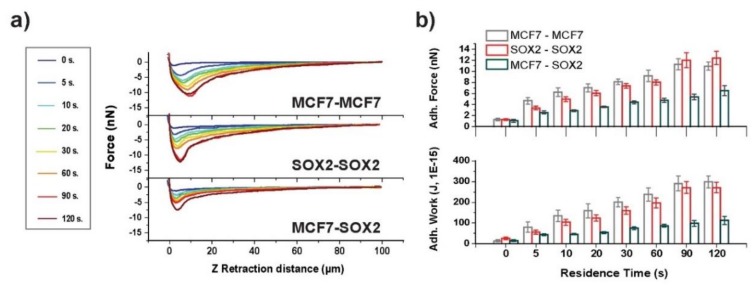
Contact time-dependent cell–cell interactions. (**a**) Contact time-dependent averaged force–distance plots for symmetric (MCF7–MCF7 and Sox2–Sox2), and the asymmetric (MCF7–Sox2) interactions for cells on top of fibronectin. (**b**) Adhesion force and work of adhesion values for the three systems under analysis.

Force–distance curves for both symmetric and asymmetric interactions between cells were analyzed (Figure 5a). An increase as a function of contact time between cells was observed, similar to the analysis of cell–substrate adhesion, again following a linear trend after 5 s of contact between cells. The adhesion peak for the Sox2–Sox2 system was sharper in comparison to the broad MCF7–MCF7 adhesion peak. While no significant differences were present for the two symmetric systems, Sox2–Sox2 cell adhesion appeared to be lower at contact times from 5 to 60 s and after that they were higher than for the MCF7–MCF7 cell interaction. Concerning the adhesive work, MCF7–MCF7 interaction appeared to be similar. Interestingly, the adhesion behavior between MCF7–Sox2 cells was very different: Both force and work were significantly lower compared to the symmetric systems (two-fold for force and three-fold for work), suggesting that cells prefer to adhere to cells similar to themselves and, therefore, indicating that the increased expression of Sox2 affects cell adhesion.

### 3.3. Sox2 Cells Have More Focal Adhesions than MCF7 Cells

**Figure 6:**
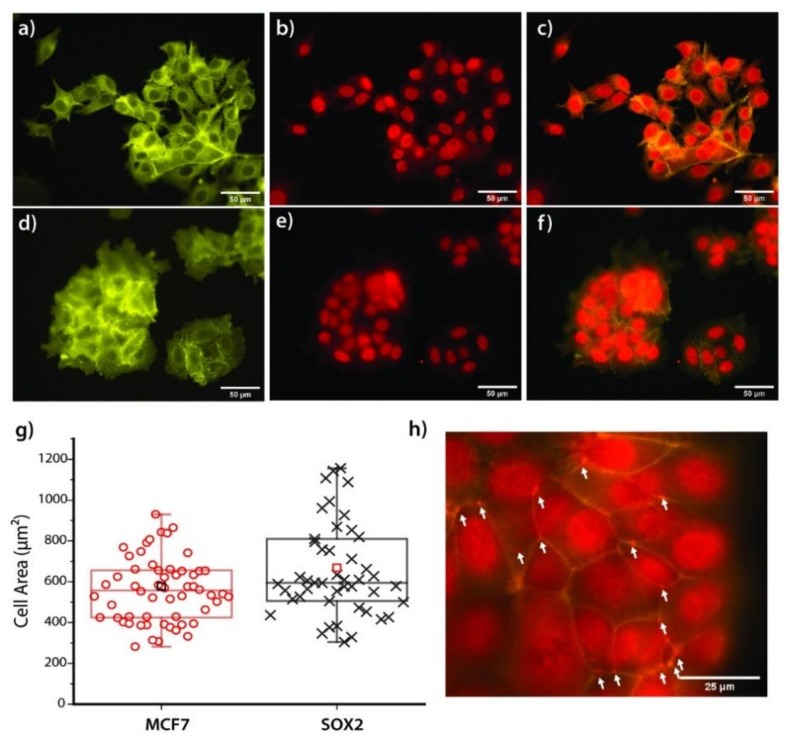
(**a**–**f**) Epifluorescence images of both MCF7 (**a**–**c**) and Sox2 (**d**–**f**) cell lines on top of Fibronectin (20 µg/mL) film upon actin (in green) and vinculin (in red) staining. The scale bars correspond to 50 µm. (**g**) Boxplot showing the distribution of the cell area values. N = 50. The square (□) shows the mean value, the horizontal line represents the median, and the values range from the 5th to the 95th quantile. (**h**) Magnification over the cell–cell connection in Sox2 cells, showing the localized agglomeration of vinculin along the contact line.

(Epi)Fluorescence microscopy was performed to evaluate the interaction of both cell lines with fibronectin (after 24 h of incubation) by staining actin and vinculin. The latter is a cytosolic protein known to be crucial for the formation of focal adhesions [41]. Generally, Sox2 cells formed large cell aggregates, while MCF7 cells still kept many individual cells and much looser cell–cell connectivity in the aggregates (Figure 6). These observations suggest that Sox2 cells present an increased affinity for fibronectin, reflected by a higher cell area (668 vs. 577 µm², Figure 6g). Actin organization along the membrane appeared to be higher for MCF7 cells (in green, Figure 6a,d). Vinculin distribution for both cell types (in red, Figure 6b,e) revealed the ubiquitous presence of the protein in both cytoplasm and nucleus, with stronger fluorescence intensity in the nucleus. Figure 6h shows the upper focal plane of Sox2 cells, merged with actin fluorescence for a better visualization. In this image, the aggregation of vinculin is observed to be aligned along cell–cell contacts.

**Figure 7:**
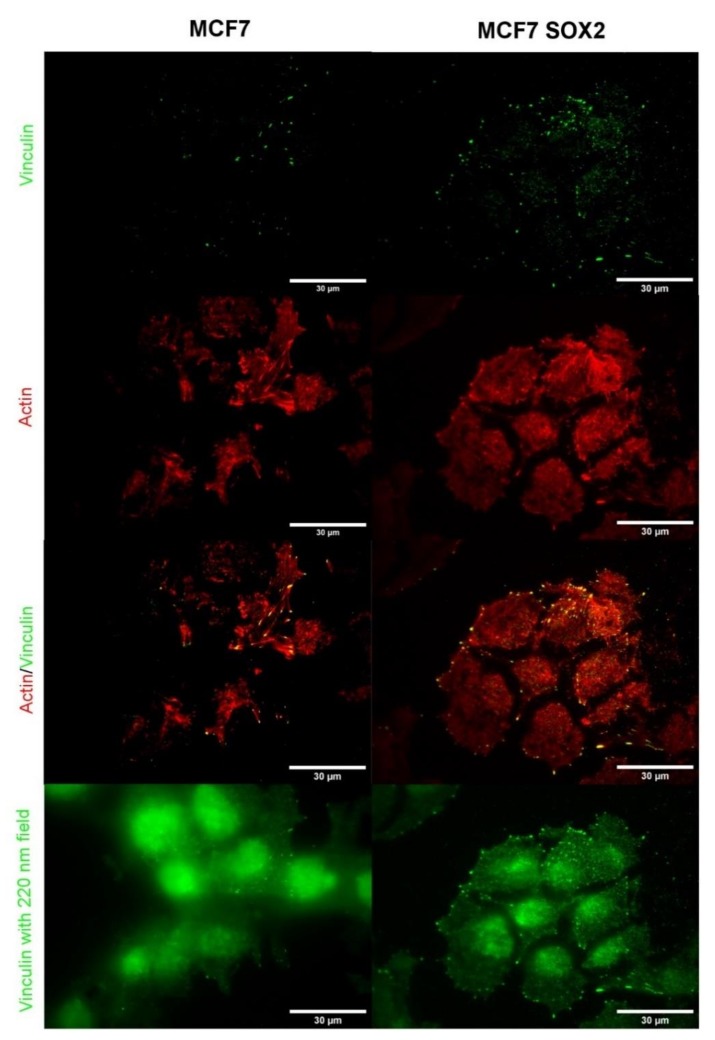
Total Internal Fluorescence Microscopy (TIRF) images of MCF7 and Sox2-overexpressing cells for individual actin (in red) and vinculin (in green) channels, and the merged image. An evanescent field of 90 nm was used while keeping the illumination time constant. The bottom image shows a wide (220 nm) field applied on the same area of interest.

In order to evaluate the formation of focal adhesions at the cell–surface interface level, TIRF microscopy was performed (Figure 7). With a TIRF field to 90 nm, the contact region of cell to substrate was imaged. Sox2 overexpressing cells seemed to form higher amount of focal adhesions, together with a higher contact area of cell to substrate, than MCF7 cells. Focal adhesions appeared both at cell edges and at central body positions. Vinculin expression was analyzed using a wider TIRF field, therefore enabling a correlation of cell shape with focal adhesion and actin stress fiber position. The ends of actin stress fibers were mostly co-localized with large focal adhesion complexes for both cell lines. MCF7 cells displayed mostly aligned F-actin bundles, while the cytoskeleton appeared more disorganized in Sox2-overexpressing cells, with actin filaments displaying several orientations. 

## 4. Discussion

Mutations and changes in protein expression lead to distinct phenotypes in tumor cells, affecting cell adhesion and mechanics, survival, proliferation, and motility. These altered adhesion properties are linked to cancer dissemination and thus worse prognosis and clinical outcome. In breast cancer patients, mortality is mostly due to the development of metastasis at specific sites, particularly bone, lung, liver, and brain tissue, each with a unique microenvironment [42]. Substantial evidence suggests that breast cancer initiation and the development of resistance and metastasis are driven by CSCs. The embryonic stem cell factor Sox2, implicated in development, pluripotency, and cancer biology [43], is also found in breast stem/progenitor cells [30] and is highly expressed in breast tumors that have developed resistance to endocrine therapy and display poor clinical outcome [29]. Furthermore, the silencing of the Sox2 gene reduces the size of the stem/progenitor cell population and restores sensitivity to tamoxifen, while the ectopic expression of Sox2 increases the CSC population and leads to reduced therapy sensitivity in vitro and in vivo [29]. Here, we assessed the adhesion behavior of Sox2-expressing cells and parental MCF7 breast cancer cells at the single cell level using AFM and fluorescence microscopy. We show that the overexpression of Sox2 leads to significant changes in adhesion both with respect to cell–substrate as well as cell–cell interactions.

The employment of single-cell probe force experiments enables the measurement of cell–surface and cell–cell interactions, based on individual cell capture and manipulation [16]. Experiments can be performed by the establishment of contact between the probe and the sample at a pre-defined load and approaching speed (factors of critical impact on the outcome observed). Due to the experimental design employed, based on pre-coated half slides, cells can be placed on top of different samples without any sample replacement being required. This is one of the stronger points in comparison to other setups.

The affinity of both MCF7 and Sox2 overexpressing cells towards non-fouling SbpA layers and specifically recognized Fibronectin (FN) films was measured in terms of both maximum adhesion and work of adhesion. The latter relates to the energy required for breaking the contact equilibrium, which might be induced by variations in the cell spreading/wetting of each system or by a tighter membrane–cytoskeleton connection. Our results (Figure 2) confirm both the effectiveness of the SbpA layer against cell binding, and the establishment of residence time-dependent and specific interactions between cells and the underlying FN film. Such specificity has been tested by the external addition of soluble Fibronectin, which effectively suppressed the previously seen cell–FN interactions (Figure 4), by blocking the specific recognition of FN by membrane receptors.

Increased Sox2 expression has been found in different types of cancer, including breast, colorectal, or lung, among others [44,45,46]. Breast tumors that have developed resistance to hormone therapy display increased Sox2 expression, which supports its potential as a biomarker of resistance to therapy. Sox2 has been implicated in tumor initiation, epithelial-to-mesenchymal transition (EMT), and resistance to therapy and metastasis in several human cancers [47]. We, therefore, hypothesized that the overexpression of Sox2 could lead to an altered phenotype with respect to cell–surface and cell–cell adhesion, as observed in other cells undergoing EMT [48]. Indeed, cells with increased levels of Sox2, which are more invasive [30], were found to display increased adhesion force. In line with these findings, Smolyakov and colleagues, using AFM, found that while adhesion to fibronectin did not lead to any significant changes, an increase in the cell–cell maximum adhesive force for the most invasive breast cancer cell lines (i.e., MDA-MB-231) was observed [49]. Similarly, Pawlizak et al. compared MCF10A, MDA-MB-231, and MDA-MB-436 breast cancer cells and confirmed the increased maximum cell–cell adhesive force for the most aggressive cancer cells [50]. Contrarily, Omidvar et al. reported a decrease in adhesion force for the most invasive breast cancer cells (MCF7 vs. T47D vs. MDA-MB-231) [51]. However, the reduction in cell–cell adhesion was claimed to be partly due to the loss of E-cadherin expression and the enhanced expression of N-cadherins, which is not expressed in these cells, as reported by Smolyakov and colleagues [49], suggesting that other adhesion receptors may be implicated. Sox2 has also been shown to affect cell–ECM interactions in other systems, for example, cell adhesion- and ECM-related genes were found to be significantly up-regulated upon Sox2 overexpression in Schwann cells, and Sox2 was identified as a key regulator of ECM remodeling during Schwann cell clustering, directional migration, and axonal guidance in vitro [52].

Symmetric cell–cell adhesion was similar for both cell types, but asymmetric measurements show a decrease of adhesion strength by a factor of 2 to 3. This significant difference likely reflects heterotypic adhesion, suggesting that the two cell lines express different surface adhesion molecules. However, this type of mixed recognition is likely to occur in vivo since intra-tumor heterogeneity has been extensively described in breast cancer [53]. Different cellular and molecular mechanisms account for tumor heterogeneity, which complicate diagnosis and prognosis and challenges cancer therapies. In fact, significant adhesion strength heterogeneity has also been observed in different cancer cell lines [54]. It has been proposed that the heterogeneous nature of breast tumors is a function of their CSC content [55]. Some reports have found that tumor cells are stiffer than normal cells [56], while others have shown that alterations of the cytoskeletal structure from an organized to an irregular network, as induced by Sox2 overexpression, reduces cell stiffness [57], supporting the view that invasive cells are softer than other cancer cells. Interestingly, circulating tumor cells (CTCs), display an increased ability to seed metastasis when found as clusters held together by intercellular junctions. These CTC clusters are particularly enriched in stem-cell-related genes, including Sox2 [58], providing another example of common features between stem biology and cancer metastasis, which led the authors to speculate that the high expression of cell–cell junction components in cancer cells may facilitate intravasation into the bloodstream as clusters, while maintaining stem-like features to enable the initiation of metastasis. Similarly, our findings have detected strong vinculin-supported lateral interactions at the cell–cell level inducing the formation of stable cell aggregates in Sox2 overexpressing cells. Furthermore, the presence of dormant tumor cells has been associated with increased metastatic risk and poor prognosis, and these cells appear to display an adherent phenotype and increased glycoproteins and proteoglycans, including fibronectin deposition, under cellular stress [59]. It will be noteworthy to further understand the mechanism by which alterations in integrin expression or syndecan levels may affect binding to fibronectin and the potential consequences in breast cancer cells.

In conclusion, the overexpression of the stem cell factor Sox2 significantly alters both cell–substrate as well as cell–cell adhesion. Further studies of the molecular mechanisms underlying these observations are warranted in the future, including 3D cultures and analysis of the influence of the extracellular matrix, which is emerging as a critical regulator of tumor cell features and behavior. 

## Supplementary Materials

The following are available online at https://www.mdpi.com/2073-4409/9/4/935/s1, Figure S1: title, Table S1: title, Video S1: title.
