# Supplementary material for: Single-Cell Probe Force Studies to Identify Sox2 Overexpression-Promoted Cell Adhesion in MCF7 Breast Cancer Cells
Source: Cells. 2020 Apr 10;9(4):935. doi: 10.3390/cells9040935 (PMC7227807; doi:10.3390/cells9040935)
Supplement: Supplementary file 1 [file cells-09-00935-s001.pdf]

cells

MDPI

Supplementary Information

Single-Cell Probe Force studies to identify Sox2 Overexpression-Promoted Cell Adhesion in MCF7 Breast Cancer Cells

Jagoba Iturri [1,*], Andreas Weber [1], María dM. Vivanco [2,*] and José L. Toca-Herrera [1]

[1] Department of Nanobiotechnology (DNBT), Institute for Biophysics, BOKU University for Natural Resources and Life Sciences, Muthgasse 11 (Simon Zeisel Haus), A-1190 Vienna, Austria; andreas.weber@boku.c.at (A.W.); jose.toca-herrera@boku.ac.at (J.L.T.-H.)

[2] Cancer Heterogeneity Lab, Center for Cooperative Research in Biosciences (CIC bioGUNE), Basque Research and Technology Alliance (BRTA), Bizkaia Technology Park, 48160 Derio, Spain

* Correspondence: jagoba.iturri@boku.ac.at (J.I.); mdmvivanco@cicbiogune.es (M.dM.V.)

Received: 20 March 2020; Accepted: 9 April 2020; Published: date

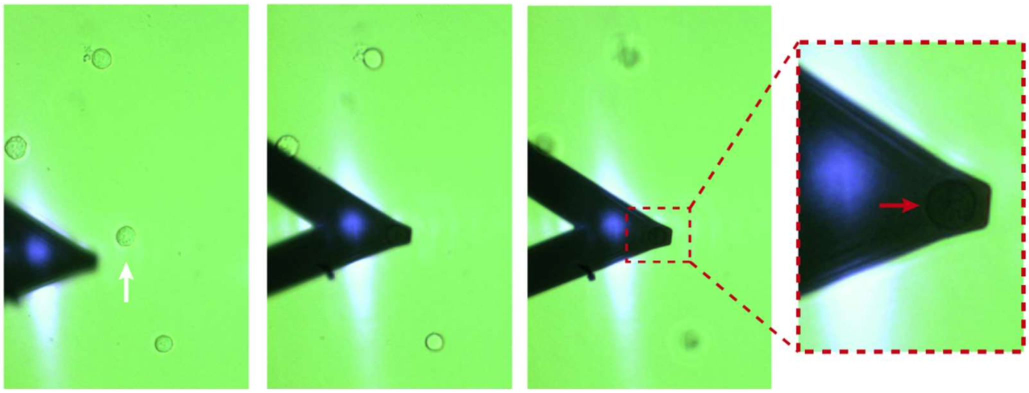

Figure S1. From left to right, sequence of the cell capture from the SbpA-coated side. A target cell (left, indicated by the white arrow) is approached and softly pressed in between the fibronectin-coated cantilever and the SbpA layer underneath by controlling the loading force. After ca. 30 seconds (right) the cantilever is retracted and the cell remains attached. The zoom-in confirms the presence of the intact cell at the end of the process.

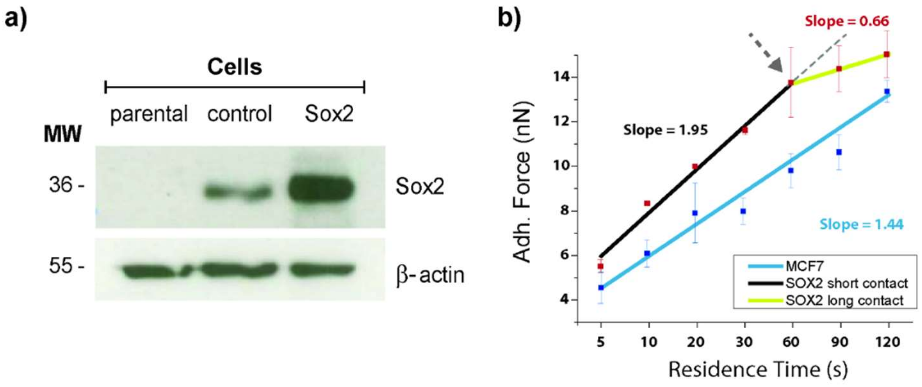

Figure S2. (**a**) Representative western blot analysis of Sox2 expression in parental MCF7 cells (parental), MCF7 cells resistant to tamoxifen as positive control for Sox2 expression [Ref 29] (control) and Sox2 overexpressing MCF7 cells (Sox2); beta-actin was used as a loading control. The molecular

weight (MW) markers are indicated. (**b**) Linear fitting for the Adhesion force values in the residence time range 5-120 s. The arrow highlights the slope switching occurring above 60 s.

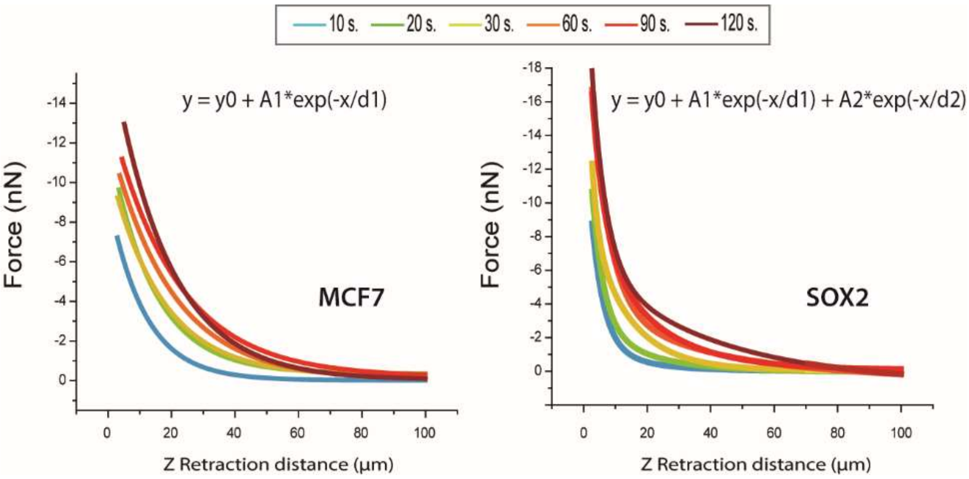

Figure S3. Residence time (10-120 s) dependent post-adhesion peak Force decay trend (single vs double exponential) calculated for the respective cell types. (See table S3 for detailed value information).

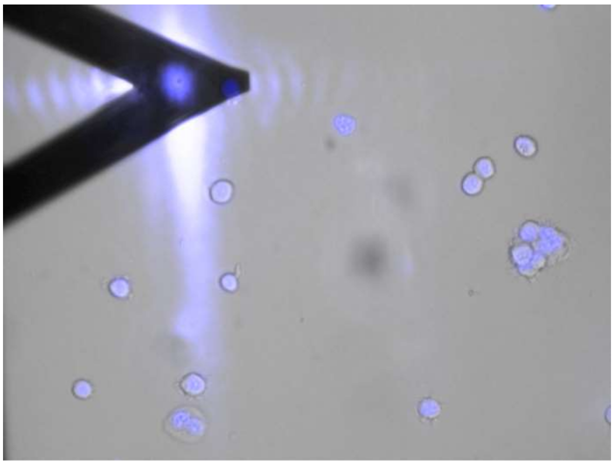

Figure S4. Merged optical and fluorescence micrographs showing the nuclei staining of Sox2 cells.

Table S1. Adhesion Force and Work values for Cell-fibronectin interactions. Force values appear normalized to cell contact radius (R = 5.3 μm).

	**Adhesion Force (nN) / R**		**Adhesion Work (J, 1E–15)**	
	**MCF7**	SOX2	MCF7	SOX2
0	0.12 ± 0.06	0.07 ± 0.01	1.2 ± 0.3	0.77 ± 0.2
5	0.89 ± 0.15	1.04 ± 0.07	40.6 ± 6.6	22.4 ± 6.9
10	1.16 ± 0.14	1.59 ± 0.02	86.2 ± 13.0	53.4 ± 13.7
20	1.56 ± 0.29	1.90 ± 0.02	150.9 ± 37.5	76.1 ± 16.2
30	1.58 ± 0.13	2.22 ± 0.26	173.0 ± 17.9	113.0 ± 21.9
60	1.90 ± 0.17	2.56 ± 0.38	222.3 ± 9.1	139.4 ± 62.6
90	2.05 ± 0.17	2.67 ± 0.24	246.0 ± 37.9	155.6 ± 62.9
120	2.58 ± 0.11	2.81 ± 0.25	288.0 ± 0.3	189.3 ± 70.6

Table S2. Exponential decay fitting values obtained from 1st and 2nd order equations for MCF7 and Sox2 cells, respectively. Adjusted R-square reflects the goodness of the fitting. The highlighted value indicates an outlier.

		1st Order		2nd Order		
	**Res. Time (s)**	**A1 ± SD**	**d1 ± SD (μm)**	**A2 ± SD**	**d2 ± SD (μm)**	**Adj. R-Square**
SOX2	10	-48.53 ± 0.39	1.04 ± 0.01	-3.52 ± 0.02	7.67 ± 0.03	0.99349
	20	-35.67 ± 0.10	1.63 ± 0.01	-1.99 ± 0.01	5.28 ± 0.05	0.99705
	30	-3.80 ± 0.02	14.82 ± 0.05	-30.23 ± 0.08	2.25 ± 0.01	0.99673
	60	-1.97 ± 0.03	71.70 ± 2.18	-34.09 ± 0.08	2.43 ± 0.01	0.99624
	90	-5.31 ± 0.06	11.36 ± 0.09	-26.32 ± 0.09	2.58 ± 0.01	0.99461
	120	-5.38 ± 0.03	15.51 ± 0.08	-39.91 ± 0.18	2.22 ± 0.01	0.99531
MCF7	10	-9.33 ± 0.01	11.32 ± 0.01			0.9957
	20	-11.86 ± 0.02	14.00 ± 0.03			0.9802
	30	-10.84 ± 0.01	16.34 ± 0.03			0.9831
	60	-12.49 ± 0.01	19.37 ± 0.03			0.9917
	90	-13.57 ± 0.01	20.96 ± 0.04			0.9865
	120	-17.21 ± 0.01	17.86 ± 0.02			0.9945

Table S3. Adhesion Force and Work values before (L15) and after (BulkFN) injection of soluble fibronectin in the measuring medium for both MCF7 and Sox2 cells.

		MCF7		SOX2	
	**Res. Time (s)**	**L15 ± SD**	**BulkFN ± SD**	**L15 ± SD**	**BulkFN ± SD**
Adh. Force	30	8.36 ± 0.69	1.42 ± 0.16	11.79 ± 0.26	2.09 ± 0.61
	60	10.09 ± 0.89	1.75 ± 0.04	13.56 ± 2.05	2.79 ± 0.58
	120	13.67 ± 0.57	2.08 ± 0.25	14.89 ± 1.33	2.67 ± 0.32
Adh. Work	30	173.5 ± 17.9	19.2 ± 1.1	113.0 ± 21.9	11.5 ± 1.9
	60	222.3 ± 9.1	31.9 ± 0.9	139.4 ± 62.6	13.1 ± 1.2
	120	288.5 ± 0.3	37.4 ± 2.3	189.3 ± 70.6	14.7 ± 1.7

Table S4. Adhesion Force and Work values for Cell-Cell interactions. Force values appear normalized to cell contact radius (R = 5.3 μm).

	**Adhesion Force (nN)**			Adhesion Work (J, 1E-15)		
	**MCF7**	**SOX2**	**Assym.**	MCF7	SOX2	Assym.
Res. Time (s)	Mean ± SEM	Mean ± SEM	Mean ± SEM	Mean ± SEM	Mean ± SEM	Mean ± SEM
0	0.24 ± 0.04	0.24 ± 0.03	0.19 ± 0.05	13.9 ± 5.3	25.3 ± 6.6	15.0 ± 3.7
5	0.88 ± 0.11	0.64 ± 0.06	0.48 ± 0.04	79.8 ± 25.2	56.5 ± 10.0	44.0 ± 4.4
10	1.17 ± 0.14	0.94 ± 0.08	0.54 ± 0.04	135.5 ± 27.8	103.9 ± 14.6	46.0 ± 4.3
20	1.32 ± 0.14	1.14 ± 0.09	0.67 ± 0.03	159 ± 34.4	124.0 ± 14.7	53.7 ± 4.9
30	1.53 ± 0.09	1.39 ± 0.08	0.83 ± 0.05	200.9 ± 21.5	160.4 ± 18.4	75.2 ± 7.1
60	1.73 ± 1.05	1.51 ± 0.08	0.90 ± 0.07	238.1 ± 32.1	196.9 ± 23.8	87.0 ± 6.8
90	2.11 ± 0.19	2.26 ± 0.24	1.01 ± 0.09	290.4 ± 35.9	270.8 ± 28.6	98.2 ± 14.2
120	2.06 ± 0.13	2.34 ± 0.23	1.22 ± 0.17	298.4 ± 28.6	271.5 ± 25.2	112.9 ± 19.5

© 2020 by the authors. Licensee MDPI, Basel, Switzerland. This article is an open access article distributed under the terms and conditions of the Creative Commons Attribution (CC BY) license (http://creativecommons.org/licenses/by/4.0/).
